# Prognostic Value of Systemic Immune-Inflammation Index among Critically Ill Patients with Acute Kidney Injury: A Retrospective Cohort Study

**DOI:** 10.3390/jcm11143978

**Published:** 2022-07-08

**Authors:** Lan Jia, Chen Li, Xueqing Bi, Fang Wei, Jia Meng, Guijiang Sun, Haibo Yu, Hongye Dong, Bo Li, Yueqi Cao, Lihua Wang, Aili Jiang

**Affiliations:** 1 Department of Kidney Disease and Blood Purification, Institute of Urology & Key Laboratory of Tianjin, The Second Hospital of Tianjin Medical University, Tianjin 300211, China; lanj1989@163.com (L.J.); bxqkwan@163.com (X.B.); weifang9468@sina.com (F.W.); gnipahellir@163.com (J.M.); sunguijiang@tmu.edu.cn (G.S.); yuhaibo2001@126.com (H.Y.); donghy110119@163.com (H.D.); libo1985@tmu.edu.cn (B.L.); onecyq@126.com (Y.C.); 2 Department of Orthopaedics, Tianjin Hospital, Tianjin 300211, China; lichen8848@126.com

**Keywords:** systemic immune-inflammation index, acute kidney injury, mortality, intensive care unit

## Abstract

Inflammation plays a significant role in the occurrence and development of acute kidney injury (AKI). Evidence regarding the prognostic effect of the systemic immune-inflammation index (SII) in critically ill patients with AKI is scarce. The aim of this study was to assess the association between SII and all-cause mortality in these patients. Detailed clinical data were extracted from the Medical Information Mart for Intensive Care Database (MIMIC)-IV. The primary outcome was set as the in-hospital mortality. A total of 10,764 AKI patients were enrolled in this study. The restricted cubic splines analyses showed a J-shaped curve between SII and the risk of in-hospital and ICU mortality. After adjusting for relevant confounders, multivariate Cox regression analysis showed that both lower and higher SII levels were associated with an elevated risk of in-hospital all-cause mortality. A similar trend was observed for ICU mortality. In summary, we found that the SII was associated in a J-shaped pattern with all-cause mortality among critically ill patients with AKI. SII appears to be have potential applications in the clinical setting as a novel and easily accessible biomarker for predicting the prognosis of AKI patients.

## 1. Introduction

Acute kidney injury (AKI) is a serious clinical condition and is associated with high morbidity and mortality, especially for critically ill patients [1,2]. The prevalence of the patients admitted to intensive care unit (ICU) can sometimes exceed 50% [3] and the mortality of these patients is as high as 60% [4]. Furthermore, an important percentage of surviving patients develops chronic kidney disease (CKD) or end-stage renal disease and needs renal replacement therapy (RRT) [5,6,7]. Considering the short and long-term effects of AKI, early identification of patients at high risk of poor prognosis is crucial for preventing complications and to decrease mortality.

Inflammation plays a significant role in the occurrence and development of AKI [8,9,10]. Many well-known inflammatory markers, including neutrophils [11], platelets [12,13] and procalcitonin [14] have been shown to be related to AKI. However, focusing on just one indicator is of limited use, as these biomarkers are always affected by other confounding factors. Nowadays, the systemic immune-inflammation index (SII) has been proposed, which is a simple and comprehensive marker. Based on PLT, neutrophils and lymphocytes, SII has the advantage of low cost and convenience and can simultaneously reflect the inflammatory and immune status of patients [15]. Its role in predicting clinical outcomes in cardiovascular disease [16,17], malignancy survival [15,18] and the incidence of contrast-induced acute kidney injury (CI-AKI) [19] has been demonstrated. Despite these observations, to the best of our knowledge, no epidemiological study to date has explored the prognostic effect of the SII in critically ill patients with AKI. The aim of this study was to assess the association between SII and mortality in these patients.

## 2. Materials and Methods

### 2.1. Data Source

This is a retrospective study using The Medical Information Mart for Intensive Care Database (MIMIC)-IV (Version 1.0) [20]. MIMIC-IV is a large, freely-available database comprising clinical data of patients admitted to the Beth Israel Deaconess Medical Center (BIDMC) between 2008 and 2019. To apply for permission to access the database, the author Lan Jia has completed the recognized course in Protecting Human Research Participants (certification number: 33918192).

### 2.2. Study Population

**Figure 1:**
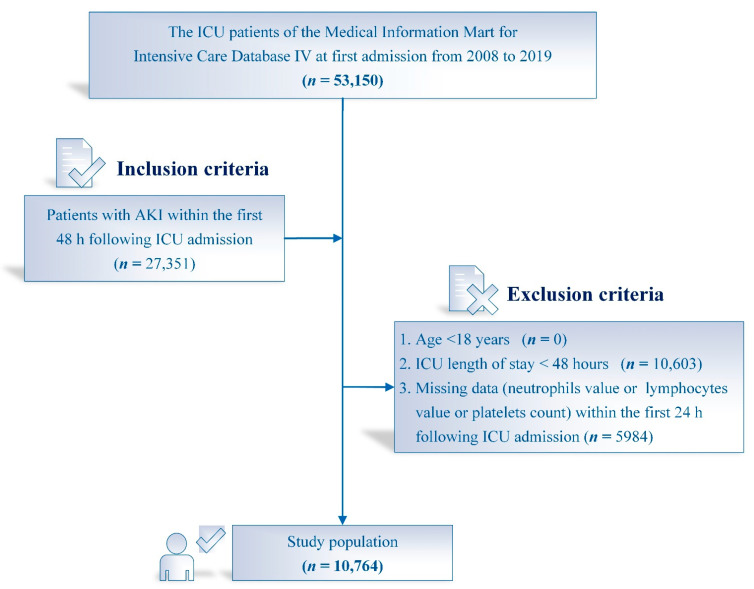
Flowchart of patient selection. *n*, Number; ICU, Intensive care unit; AKI, Acute kidney injury.

A total of 53,150 ICU patients at first admission were recorded in the MIMIC-IV. The flowchart of this study and the number of patients is presented in Figure 1. Only patients diagnosed with AKI were included. The exclusion criteria were as follows: (1) age <18 years; (2) length of stay in ICU < 48 h; (3) missing platelets count or neutrophils or lymphocytes values at ICU admission.

The classification of AKI was determined on the basis of Kidney Disease Improving Global Outcomes (KDIGO) [21]. AKI was identified by reduced urine output (urine volume < 0.5 mL/kg/h for ≥6 h) and increased serum creatinine (SCr) level (an increase in SCr of ≥0.3 mg/dL within 48 h or an increase in SCr to ≥1.5 times baseline within 7 days). Stage 1 was defined as an increase in SCr to ≥1.5 times baseline or an increase in SCr by ≥0.3 mg/dL or urine output <0.5 mL/kg/h for 6 to 12 h. Stage 2 was defined as an increase in SCr to ≥2.0 times baseline or urine output <0.5 mL/kg/h for ≥12 h. Stage 3 was defined as an increase in SCr to ≥3.0 times baseline or SCr value ≥4.0 mg/dL or initiation of RRT or urine output <0.3 mL/kg/h for ≥24 h or anuria for ≥12 h. AKI stage was determined by SCr and urine output within the first 48 h following ICU admission.

### 2.3. Date Extraction

Patient data within the first 24 h after ICU admission was extracted from MIMIC-IV using Structured Query Language (SQL) and was collected as follows: (1) Comorbidities: atrial fibrillation, coronary artery disease (CAD), congestive heart failure (CHF), hypertension, diabetes mellitus, chronic obstructive pulmonary disease (COPD), respiratory failure, CKD, stroke, chronic liver disease, hepatic failure and malignant tumor; (2) Vital signs: heart rate, mean blood pressure (MBP), respiratory rate, temperature and percutaneous oxygen saturation (SPO2); (3) Laboratory parameters: hemoglobin, white blood cell (WBC) count, platelets count, SCr, blood urea nitrogen (BUN), serum bicarbonate, serum sodium, serum potassium, serum glucose and serum lactate; (4) Scoring systems of severity-of-illness: sequential organ failure assessment (SOFA) score, simplified acute physiology score II (SAPSII) and Glasgow coma scale (GCS) score. Vasopressors included epinephrine, norepinephrine, dopamine, dobutamine and vasopressin.

### 2.4. Definition of SII and Study Endpoint

The primary outcome was set as the in-hospital mortality. The secondary outcome was ICU mortality. Survival data was extracted from MIMIC-IV. The SII was calculated using the following equation: platelets × neutrophils/lymphocytes [15].

### 2.5. Missing Data

In this study, the missing values of all variables were less than 20% and we used multiple imputation [22] to impute missing values of variables.

### 2.6. Statistical Analysis

The baseline characteristics of all patients were stratified by SII quintile. Continuous variables were described as the medians and interquartile range (IQR) and were compared using the Kruskal–Wallis test. Categorical variables were described as frequencies or percentages and were compared using Chi-square. Cox proportional hazards models were performed to identify the association between the SII and the study endpoint and the results were presented as hazard ratio (HR) with 95% confidence intervals (CIs). We also used restricted cubic splines to identify the association between SII and all-cause mortality in critically ill patients with AKI.

For each outcome, three multivariate models were constructed based on SII quintiles. Covariates in Model 1 were adjusted for age, gender and ethnicity. Model 2 included Model 1 and comorbidities such as atrial fibrillation, CAD, CHF, hypertension, diabetes mellitus, COPD, respiratory failure, CKD, stroke, chronic liver disease, hepatic failure, malignant tumor and AKI stage. In Model 3, we further adjusted for MBP, heart rate, respiratory rate, temperature, SPO2, SAPSII, SOFA, GCS, hemoglobin, WBC count, SCr, BUN, serum bicarbonate, serum sodium, serum potassium, serum glucose, lactate and vasopressors use.

Stratification analyses were performed to determine the relationship between the SII and in-hospital mortality across various subgroups, such as age, gender, atrial fibrillation, CAD, CHF, hypertension, diabetes mellitus, COPD, respiratory failure, CKD, stroke, chronic liver disease, hepatic failure, malignant tumor, SOFA, SAPSII, MBP, heart rate, WBC count, SCr, BUN, lactate, AKI stage, vasopressors use. All statistical analyses were performed with the software Stata software (Version 17.0, StataCorp LLC, College Station, TX, USA) and R software (Version 4.1.3, R Foundation for Statistical Computing, https://www.r-project.org/, accessed on 10 May 2022). A two-tailed *p* value < 0.05 was considered statistically significant.

## 3. Results

### 3.1. Patient Characteristics

The study flow chart is shown in Figure 1. In total, 10,764 AKI patients were eligible for the study according to the inclusion and exclusion criteria, including 6135 men (57%) and 4629 women. The median age and the SII level were 69.1 years (57.5–79.7) and 1519.8 × 10^9^/L (700.1–3201.5), respectively. Table 1 summarizes the characteristics of the study cohorts stratified by SII. AKI patients were divided into five groups according to SII quintiles. Patients with higher SII values were more likely to be elderly, female and white, with a history of chronic heart failure, respiratory failure and malignant tumor. They also had higher levels of white blood cells, platelets, serum creatinine, serum urea nitrogen, serum glucose and serum lactate. Patients with higher SII levels were more likely to be diagnosed at a higher stage of AKI and to have received RRT than those with lower SII. Furthermore, patients with higher SII values had higher SAPSII scores, use of vasopressors and longer length of ICU stay and hospital stay.

### 3.2. Association between SII and Outcomes

**Figure 2:**
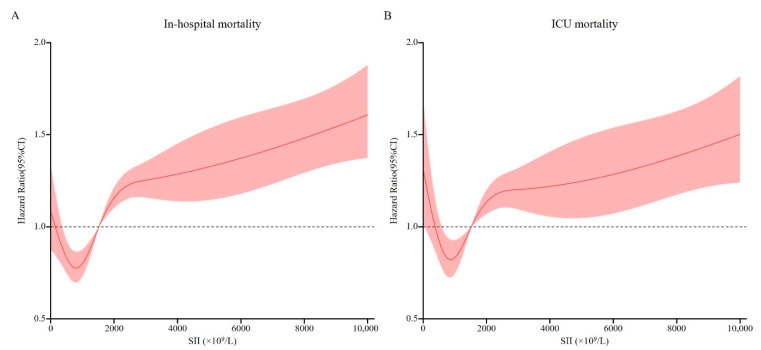
Relationship between the SII and the risk of all-cause mortality. (**A**) Relationship between the SII and in-hospital mortality. (**B**) Relationship between the SII and the ICU mortality. Shaded areas around the curves depict 95% confidence interval. SII, Systemic-immune inflammation index; ICU, Intensive care unit.

We used restricted cubic splines to flexibly model and visualize the relationship between SII and in-hospital and ICU mortality. As shown in Figure 2, there was a nonlinear association between SII and in-hospital mortality. Regarding a J-shaped relation between SII and in-hospital mortality, the plot showed a reduction of the risk within the lower range of SII, which reached the lowest risk around 804.02 × 10^9^/L and then started to increase rapidly afterwards (*p* for non-linearity <0.001). A similar trend was observed for ICU mortality, as shown in Figure 2. A J-shaped curve was observed, demonstrating that both lower and higher SII level were associated with an elevated risk of ICU all-cause mortality.

A total of 1862 in-hospital deaths and 1301 ICU deaths occurred during follow-up. We used Cox proportional hazards regression models to determine the association between SII and in-hospital and ICU all-cause mortality in patients with AKI (Table 2). In Model 1, after adjusting for age, sex and ethnicity, the adjusted HR (95%CIs) for the first, third, fourth and fifth quintiles were 1.19 (1.00–1.40), 1.39 (1.18–1.64), 1.54 (1.32–1.81) and 1.86 (1.60–2.16), respectively, and the second quintile had the lowest risk. In Model 3, after adjusting for Model 2 and other relevant confounders such as vital signs, scoring system and laboratory data, we established that higher SII remained significantly associated with an increased risk of in-hospital mortality; adjusted HR (95% CIs) for the third, fourth and fifth quintiles were 1.33 (1.13–1.57), 1.40 (1.19–1.64) and 1.49 (1.27–1.75), respectively. Marginally increased risk was associated with extremely low SII (≤582.2; *p* = 0.0831). A similar trend was observed for ICU mortality, as shown in Table 2.

### 3.3. Subgroup Analyses

**Figure 3:**
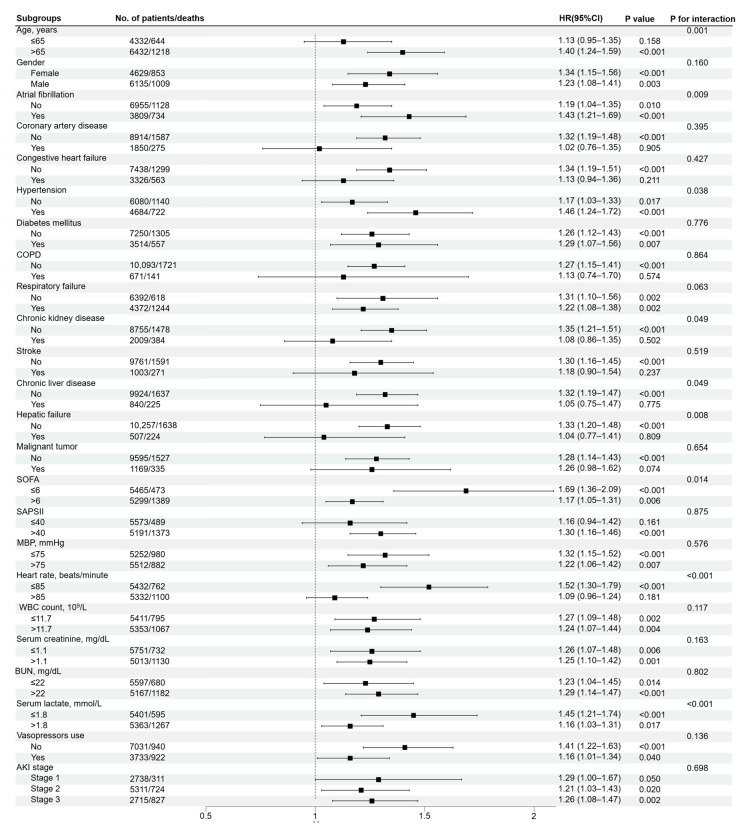
Forest plot of SII for in-hospital mortality in prespecified subgroups. Multivariable Cox regression analysis in subgroups adjusted the same covariates as for Model 3 in Table 2. SII, Systemic immune-inflammation index; COPD, Chronic obstructive pulmonary disease; MBP, Mean blood pressure; WBC, White blood cell; SCr, Serum creatine; BUN, Blood urea nitrogen; SOFA, Sequential organ failure assessment; SAPSII, Simplified acute physiology score II; AKI, Acute kidney injury.

We conducted subgroup analyses to determine the association between SII and in-hospital mortality across comorbidities and different parameters, as shown in Figure 3. In the adjusted Model 3, the results showed that increase of SII levels was related to the rise of in-hospital all-cause mortality among critically ill patients with AKI in most of the sub-populations. In addition, most of the stratification factors were not found to contribute to the association between SII and in-hospital mortality, except for a significant interaction observed for age (*p* = 0.001), atrial fibrillation (*p* = 0.009), hypertension (*p* = 0.038), CKD (*p* = 0.049), chronic liver disease (*p* = 0.049), hepatic failure (*p* = 0.008), SOFA (*p* = 0.014), heart rate (*p* < 0.001) and lactate (*p* < 0.001).

## 4. Discussion

In this study, we observed a J-shaped association between SII and in-hospital and ICU mortality. In the fully adjusted model, we found that both lower and higher SII level were associated with an elevated risk of all-cause mortality. Therefore, SII may be a useful new biomarker for predicting the prognosis of AKI patients, which will enable early intervention and facilitate AKI risk stratification management by identifying AKI patients at risk of death or requiring RRT.

Inflammation plays a crucial role in both the initial and subsequent stages of AKI [8,9,23]. Furthermore, inflammation is also associated with AKI-to-CKD transition [24,25]. In the early stages of inflammation, neutrophils are the first cells recruited from the blood into tissues; they engulf pathogens and particles, generate reactive oxygen and nitrogen species, and release antimicrobial peptides [26]. Neutrophil infiltration was detected in the kidneys of ischemia-reperfusion injury (IRI) rats [27] and in biopsy samples from patients with early AKI [28,29]. Therefore, neutrophils play an important role in the pathogenesis of kidney injury. Lymphocytes, however, are negatively correlated with inflammation, and the reduction of lymphocytes leads to abnormal immune function, which promotes the development of renal injury [30]. Fisher et al. [31] found that neutrophil-lymphocyte ratio (NLR) was an independent predictor of severe AKI (stages 2 and 3) in patients with COVID-19, which was useful for risk stratification to predict severe AKI. In addition, in a study of 1168 hospitalized patients with AKI, NLR was an independent risk factor for RRT requirement and all-cause mortality. Patients with NLR ≥5.51 suffered 1.7-fold risk of RRT requirement and 2.5-fold risk of death, compared with patients with low NLR after adjusting for multiple potential confounding factors [32]. In conclusion, the imbalance of inflammatory cells, especially the increase of neutrophils and the decrease of lymphocytes, plays an important role in the occurrence and development of AKI.

Accumulating evidence suggests that platelets play a significant role in the pathology of AKI [12]. During the process of inflammation, free platelets could bind to neutrophils to form platelet-neutrophil aggregates (PNA) [33]. The substances released by PNA induce tissue invasion of neutrophils, release of inflammatory mediators and lead to renal injury through vascular injury and tissue destruction. Furthermore, platelets contribute to thrombus formation and induce microvascular embolism, which also plays an important role in the pathogenesis of AKI [34]. Several studies have demonstrated the relationship between platelet count and AKI [34], cardiovascular mortality [35] and malignancy survival [36]. However, none of these biomarkers could comprehensively reflect the inflammatory and immune status of body. SII integrates the predictive power of the three indicators (neutrophils, lymphocytes and platelet counts), which may potentially reflect the balance of the inflammatory, immune and thrombotic pathways. Furthermore, compared to other new biomarkers, including neutrophil gelatinase-associated lipocalin (NGAL), insulin-like growth factor-binding protein 7 (IGFBP7), tissue inhibitor of metalloprotease-2 (TIMP-2) and high mobility group box-1 (HMGB-1), SII is convenient and cost-effective [37,38,39]. Hu et al. [15] found that SII contributed to the risk stratification of hepatocellular carcinoma (HCC) patients. HCC patients with SII ≥ 330 × 10^9^/L had higher recurrence rates and shorter survival time than patients with SII < 330 × 10^9^/L. In a retrospective cohort study of 4606 critically ill patients with CHF, high levels of SII could effectively predict high 30- and 90-day and hospital mortalities, as well as the high risk of occurrence of major cardiovascular adverse events (MACEs) [16]. In a study of 4381 patients, Jiang et al. [19] demonstrated that elevated SII levels were independently associated with increased risk of CI-AKI in patients undergoing coronary angiography. Compared with patients with low SII (<300 × 10^9^/L), patients with high SII (≥1200 × 10^9^/L) had a 1.9-fold increase in the risk of developing CI-AKI (OR, 2.914; 95% CI, 2.121–4.003). Furthermore, compared with other single biomarkers (NLR, platelet and C-reaction protein), SII had the most strongly positive association with the incidence of CI-AKI. Similarly, based on the association between SII and disease severity, our findings show that higher SII is associated with poorer clinical outcomes among critically ill patients with AKI.

Neutropenia [26,40] and thrombocytopenia [41,42] are common among critically ill patients and indicate that the body may be in disorder, with problems such as serious inflammation or myelosuppression, which are usually associated with poor prognosis. In the PICARD multicenter cohort study including 618 critically ill patients with acute renal failure, Mehta et al. [43] found that hematologic failure was significantly associated with mortality (adjusted OR, 3.40; 95% CI, 2.03–5.70) and was defined as WBC ≤ 1000/mm^3^ and platelet counts < 20,000/mm^3^. Kertai et al. [34] assessed the risk factors for postoperative AKI in 4217 patients undergoing coronary artery bypass grafting surgery and demonstrated that the risk of postoperative AKI increased by 14% for every 30 × 10^9^/L reduction in platelet counts. Patients with thrombocytopenia (platelet count ≤ 74 × 10^9^/L) were 3 times more likely to progress to a higher severity of postoperative AKI (adjusted OR, 3.04; 95% CI, 2.26–4.07) and had an increased risk of mortality immediately after surgery (adjusted HR, 5.46; 95% CI, 3.79–7.89). Taken together, these findings suggest that low neutrophil counts and low platelet counts may be responsible for lower SII in patients with AKI and could contribute to increased mortality, thus helping to explain our observation of a J-shaped relationship between SII and mortality, demonstrating that patients with lower SII still had an increased risk of all-cause mortality.

In our exploratory analysis of subgroups, no significant interactions were observed for gender, CAD, CHF, diabetes mellitus, COPD, respiratory failure, stroke, malignant tumor, SAPSII, MBP, WBC count, SCr, BUN, AKI stage or vasopressors use when patients were stratified according to potential confounders. However, SII had an interaction with age, atrial fibrillation, hypertension, CKD, chronic liver disease, hepatic failure, SOFA, heart rate and lactate. SII may be valuable for the prognosis evaluation of AKI patients with atrial fibrillation or hypertension, but seemed to be of weak prognostic value for AKI patients with CKD, chronic liver disease and hepatic failure. Similarly, the previous study also showed that raised SII was associated with an increased risk of the postoperative recurrence of atrial fibrillation and independently predicted the recurrence of atrial fibrillation after cryoablation concomitant with mitral valve surgery (OR, 3.719; 95% CI, 1.417–9.760) [44]. Although there was no research on the correlation between SII and hypertension, the interconnection of inflammation and the pathogenesis of hypertension has been recognized [45,46]. In addition, patients with SOFA score ≤ 6, heart rate ≤ 85 beats/minute and lactate ≤ 1.8 mmol/L had a higher risk of all-cause mortality for higher SII. Although possible interactions between these clinical factors and SII have been observed, little is known about the mechanisms and further studies are needed to confirm their relationship.

The main strength of our study is that, to the best of our knowledge, it is the first study to investigate the relationship between SII and mortality among critically ill patients with AKI. Furthermore, the large sample size of this study increases the reliability of the results. However, this study also has some limitations. First, this is a single-center retrospective study and selection bias cannot be ignored. Second, the data were collected retrospectively and some important variables may be omitted due to insufficient data. Although we adjusted for confounders, our results may have been affected by other unknown factors. Third, we measured SII only upon admission to the ICU and did not assess the changes during hospitalization. A single measure of SII could not fully reflect the inflammatory and immune status of patients. Additionally, other inflammatory markers, such as C reactive protein or procalcitonin, are rarely used in the routine clinical diagnosis and treatment. Finally, the MIMIC-IV database does not provide long-term follow-up information and we only use in-hospital and ICU mortality, which may influence prognostic assessments.

## 5. Conclusions

In summary, we found a J-shaped relationship between SII and all-cause mortality. Both lower and higher SII were associated with an increased risk of in-hospital and ICU mortality in critically ill patients with AKI. Therefore, SII has potential applications in the clinical setting as a cost-effective and easily accessible biomarker. However, our findings need to be validated with larger prospective studies and longer follow-up.

## Figures and Tables

**Table 1:** Baseline characteristics of the study population.

Characteristics	SII					*p* Value
	SII ≤ 582.2	582.6 < SII ≤ 1132.6	1132.6 < SII ≤ 2033.5	2033.5 < SII ≤ 3888.3	SII > 3888.3	
*n*	2154	2152	2153	2153	2152	
Age, years	67.3 (57.3–77.7)	68.9 (57.4–79.5)	69.6 (58.3–79.8)	69.6 (57.1–81.1)	70.3 (58.1–80.7)	<0.001
**Gender, *n* (%)**						<0.001
Male	1278 (59.3)	1293 (60.1)	1281 (59.5)	1167 (54.2)	1116 (51.9)	
Female	876 (40.7)	859 (39.9)	872 (40.5)	986 (45.8)	1036 (48.1)	
**Ethnicity, *n* (%)**						<0.001
White	1352 (62.8)	1437 (66.8)	1390 (64.6)	1425 (66.2)	1486 (69.1)	
Black	250 (11.6)	188 (8.7)	204 (9.5)	167 (7.8)	137 (6.4)	
Asian	67 (3.1)	51 (2.4)	50 (2.3)	45 (2.1)	51 (2.4)	
Other	485 (22.5)	476 (22.1)	509 (23.6)	516 (24.0)	478 (22.2)	
**Vital signs**						
Heart rate, beats/minute	82.3 (74.4–94.0)	82.6 (74.5–93. 6)	84.4 (74.6–97.3)	85.8 (74. 9–98.3)	90.1 (78.2–102.0)	<0.001
MBP, mmHg	75.0 (69.9–81.0)	75.3 (69.9–82.0)	75.5 (70.3–83.0)	75.7 (69.9–83.3)	74.7 (69.3–82.5)	0.007
Respiratory rate, beats/minute	18.3 (16.4–21.1)	18.6 (16.5–21.2)	19.1 (17.0–21.9)	19.5 (17.3–22.6)	20.4 (17.8–23.5)	<0.001
Temperature, °C	36.8 (36.5–37.1)	36. 8 (36.6–37.1)	36.8 (36.6–37.2)	36.9 (36.6–37.2)	36.9 (36.6–37.2)	<0.001
SpO_2_, %	97.6 (96.3–98.8)	97.4 (96.0–98.7)	97.2 (95.7–98.6)	97.2 (95.6–98.6)	97.0 (95.4–98.5)	<0.001
**Comorbidities, *n* (%)**						
Atrial fibrillation	738 (34.3)	798 (37.1)	800 (37.2)	722 (33.5)	751 (34.9)	0.036
Coronary artery disease	354 (16.4)	411 (19.1)	389 (18.1)	356 (16.5)	340 (15.8)	0.025
Congestive heart failure	527 (24.5)	638 (29.7)	740 (34.4)	721 (33.5)	700 (32.5)	<0.001
Hypertension	958 (44.5)	950 (44.1)	951 (44.2)	936 (43.5)	889 (41.3)	0.215
Diabetes mellitus	649 (30.1)	734 (34.1)	720 (33.4)	718 (33.4)	693 (32.2)	0.048
COPD	123 (5.7)	130 (6.0)	142 (6.6)	122 (5.7)	154 (7.2)	0.203
Respiratory failure	712 (33.1)	697 (32.4)	853 (39.6)	991 (46.0)	1119 (52.0)	<0.001
Chronic kidney disease	372 (17.3)	412 (19.1)	403 (18.7)	402 (18.7)	420 (19.5)	0.390
Stroke	157 (7.3)	215 (10.0)	250 (11.6)	214 (9.9)	167 (7.8)	<0.001
Chronic liver disease	260 (12.1)	175 (8.1)	171 (7.9)	114 (5.3)	120 (5.6)	<0.001
Hepatic failure	128 (5.9)	98 (4.6)	99 (4.6)	103 (4.8)	79 (3.7)	0.013
Malignant tumor	189 (8.8)	155 (7.2)	199 (9.2)	251 (11.7)	375 (17.4)	<0.001
**Laboratory parameters**						
Hemoglobin, g/dL	9.9 (8.4–11.9)	10.8 (9.0–12.9)	11.4 (9.6–13.2)	11.5 (9.7–13.3)	11.1(9.5–12.9)	<0.001
WBC count, 10^9^/L	7.9 (5.4–11.4)	9.9 (7.5–13.5)	11.4 (8.5–15.2)	13.2 (10.2–17.5)	16.8 (12.5–22.0)	<0.001
Platelet count, 10^9^/L	117.0 (78.0–161.0)	167.0 (125.0–217.0)	197.0 (154.0–252.0)	230.0(179.0–291.0)	287.0 (209.0–383.0)	<0.001
Serum bicarbonate, mmol/L	23.0 (20.0–25.0)	23.0 (20.0–25.0)	23.0 (20.0–26.0)	22.0 (19.0–25.0)	22.0 (19.0–25.0)	<0.001
Serum sodium, mmol/L	139.0 (136.0–141.0)	139.0 (136.0–141.0)	139.0 (136.0–141.0)	138.0 (135.0–141.0)	138.0 (134.0–141.0)	<0.001
Serum potassium, mmol/L	4.2 (3.8–4.7)	4.2 (3.9–4.7)	4.3 (3.8–4.8)	4.3 (3.8–4.8)	4.3 (3.9–4.9)	<0.001
Serum creatinine, mg/dL	1.0 (0.8–1.5)	1.1 (0.8–1.5)	1.1 (0.8–1.7)	1.2 (0.8–1.9)	1.2 (0.8–2.0)	<0.001
BUN, mg/dL	19.0 (14.0–31.0)	20.0 (14.0–31.0)	22.0 (15.0–36.0)	24.0 (16.0–40.0)	25.0 (16.8–42.0)	<0.001
Serum glucose, mg/dL	115.0 (98.0–147.0)	121.0 (101.0–159.0)	131.0 (108.0–173.0)	141.0 (111.0–186.0)	144.0 (113.0–192.0)	<0.001
Serum lactate, mmol/L	1.7 (1.2–2.5)	1.7 (1.3–2.5)	1.8 (1.3–2.7)	1.9 (1.4–3.0)	2.1 (1.5–3.2)	<0.001
**Scoring systems**						
SAPSII	39.0 (31.0–49.0)	38.0 (31.0–48.0)	39.0 (31.0–49.0)	40.0 (32.0–50.0)	43.0 (34.0–53.0)	<0.001
SOFA	7.0 (5.0–10.0)	6.0 (4.0–9.0)	6.0 (4.0–9.0)	6.0 (4.0–9.0)	7.0 (4.0–10.0)	<0.001
GCS	14.0 (10.0–15.0)	14.0 (9.0–15.0)	13.0 (8.0–14.0)	13.0 (8.0–14.0)	13.0 (8.0–14.0)	<0.001
**Vasopressors use, *n* (%)**	751 (34.9)	697 (32.4)	724 (33.6)	727 (33.8)	834 (38.8)	<0.001
**AKI stage, *n* (%)**						<0.001
Stage 1	636 (29.5)	562 (26.1)	495 (23.0)	557 (25.9)	488 (22.7)	
Stage 2	1062 (49.3)	1125 (52.3)	1134 (52.7)	1002 (46.5)	988 (45.9)	
Stage 3	456 (21.2)	465 (21.6)	524 (24.3)	594 (27.6)	676 (31.4)	
RRT	255 (11.8)	212 (9.9)	235 (10.9)	274 (12.7)	259 (12.0)	0.033
ICU length of stay, days	3.6 (2.5–6.2)	3.8 (2.7–6.5)	4.0 (2.8–7.0)	4.3 (2.8–8.0)	4.6 (2.9–8.1)	<0.001
Hospital length of stay, days	9.1 (6.1–15.5)	9.0 (5.9–14.8)	9.6 (6.2–15.2)	10.2 (6.3–17.1)	10.9 (6.8–18.1)	<0.001

SII, Systemic immune-inflammation index; N: Number; MBP, Mean blood pressure; SPO_2_, Percutaneous oxygen saturation; COPD, Chronic obstructive pulmonary disease; WBC, White blood cell; BUN, Blood urea nitrogen; SOFA, Sequential organ failure assessment; SAPSII, Simplified acute physiology score II; GCS, Glasgow coma scale; AKI, Acute kidney injury; RRT, Renal replacement therapy; ICU, intensive care unit.

**Table 2:** The multivariate Cox regression analysis for exploring the association of SII with all-cause mortality among critically ill patients with AKI.

SII	No. of Patients/Deaths	Model 1		Model 2		Model 3	
		HR (95% CIs)	*p* Value	HR (95% CIs)	*p* Value	HR (95% CIs)	*p* Value
**In-hospital mortality**							
SII ≤ 582.2	2154/308	1.19 (1.00–1.40)	0.045	1.17 (0.99–1.39)	0.065	1.16 (0.98–1.38)	0.083
582.6 < SII ≤ 1132.6	2152/247	1(reference)		1(reference)		1(reference)	
1132.6 < SII ≤ 2033.5	2153/355	1.39 (1.18–1.64)	<0.001	1.34 (1.14–1.58)	<0.001	1.33 (1.13–1.57)	<0.001
2033.5 < SII ≤ 3888.3	2153/411	1.54 (1.32–1.81)	<0.001	1.47 (1.25–1.72)	<0.001	1.40 (1.19–1.64)	<0.001
SII > 3888.3	2152/541	1.86 (1.60–2.16)	<0.001	1.66 (1.43–1.94)	<0.001	1.49 (1.27–1.75)	<0.001
**ICU mortality**							
SII ≤ 582.2	2154/213	1.23 (1.01–1.50)	0.042	1.19 (0.98–1.45)	0.084	1.14 (0.93–1.39)	0.213
582.6 < SII ≤ 1132.6	2152/180	1(reference)		1(reference)		1(reference)	
1132.6 < SII ≤ 2033.5	2153/254	1.25 (1.03–1.51)	0.023	1.27 (1.05–1.54)	0.015	1.24 (1.02–1.51)	0.028
2033.5 < SII ≤ 3888.3	2153/288	1.34 (1.11–1.62)	0.002	1.34 (1.11–1.62)	0.002	1.24 (1.02–1.50)	0.029
SII > 3888.3	2152/366	1.60 (1.34–1.91)	<0.001	1.54 (1.28–1.84)	<0.001	1.29 (1.07–1.56)	0.009

Model 1: The covariates were adjusted for age, gender, ethnicity. Model 2: The covariates were adjusted for age, gender, ethnicity, atrial fibrillation, coronary artery disease, congestive heart failure, hypertension, diabetes mellitus, chronic obstructive pulmonary disease, respiratory failure, chronic kidney disease, stroke, chronic liver disease, hepatic failure, malignant tumor and acute kidney injury stage. Model 3: The covariates were adjusted for age, gender, ethnicity, atrial fibrillation, coronary artery disease, congestive heart failure, hypertension, diabetes mellitus, chronic obstructive pulmonary disease, respiratory failure, chronic kidney disease, stroke, chronic liver disease, hepatic failure, malignant tumor, acute kidney injury stage, mean blood pressure, heart rate, respiratory rate, temperature, percutaneous oxygen saturation, sequential organ failure assessment, simplified acute physiology score II, glasgow coma scale, hemoglobin, white blood cell count, serum creatine, blood urea nitrogen, serum bicarbonate, serum sodium, serum potassium, serum glucose, lactate and vasopressors use. SII, Systemic immune-inflammation index. AKI, Acute kidney injury; HR, Hazard ratio; CIs, Confidence intervals.

## Data Availability

The datasets used and analyzed during the current study are available from the corresponding author on reasonable request.
